# Chemical Reactivity and Spectroscopy Explored From QM/MM Molecular Dynamics Simulations Using the *LIO* Code

**DOI:** 10.3389/fchem.2018.00070

**Published:** 2018-03-21

**Authors:** Juan P. Marcolongo, Ari Zeida, Jonathan A. Semelak, Nicolás O. Foglia, Uriel N. Morzan, Dario A. Estrin, Mariano C. González Lebrero, Damián A. Scherlis

**Affiliations:** ^1^DQIAyQF, INQUIMAE-CONICET, Facultad de Ciencias Exactas y Naturales, Universidad de Buenos Aires, Buenos Aires, Argentina; ^2^Departamento de Bioquímica and Center for Free Radical and Biomedical Research, Facultad de Medicina, Universidad de la República, Montevideo, Uruguay

**Keywords:** QM/MM, DFT, GPU, free energy, TDDFT

## Abstract

In this work we present the current advances in the development and the applications of *LIO*, a lab-made code designed for density functional theory calculations in graphical processing units (GPU), that can be coupled with different classical molecular dynamics engines. This code has been thoroughly optimized to perform efficient molecular dynamics simulations at the QM/MM DFT level, allowing for an exhaustive sampling of the configurational space. Selected examples are presented for the description of chemical reactivity in terms of free energy profiles, and also for the computation of optical properties, such as vibrational and electronic spectra in solvent and protein environments.

## Introduction

The accurate prediction of the physicochemical properties of molecules within a realistic description of the surrounding environment is essentially the driving force in the development of QM/MM methods. The evolution of this field is not only associated with theoretical progresses, but is also very much related, and often constrained, by the advances in computer power. The last few decades have witnessed the birth and growth of two large categories of QM/MM strategies: those based on expensive QM levels of theory [like Density Functional Theory (DFT) or perturbation methods], but only applicable to dozens of quantum-mechanical atoms and a few nuclear configurations; and those that may account for thermal effects, i.e., thousands or even millions of configurations, affordable at the expense of a reduction in the accuracy of the electronic structure calculations, mostly by means of semiempirical approaches.

In this contribution, we present and review the current advances in the development of *LIO*, a lab-made code designed for electronic structure calculations exploiting the computational advantages of graphic processing units. This code can be coupled to molecular dynamics (MD) engines to perform QM/MM simulations, allowing for exhaustive MD sampling at the DFT level. In the next section, we provide an overview on the program structure and its capabilities, accompanied by some benchmarks illustrating efficiency. Then, we review a series of representative applications of our methodology, namely the description of chemical reactivity in terms of free energy profiles, and the prediction of optical properties, such as vibrational and electronic spectra. Closing remarks are given in the final section, along with some observations concerning current and future directions.

## The *LIO* code

*LIO* (https://github.com/MALBECC/LIO) is a highly efficient tool to solve the electronic structure problem in molecules using DFT and Time Dependent DFT (TDDFT) frameworks. *LIO* is designed to be used as a standalone application or as a library to be combined with other molecular dynamics packages, to run QM/MM simulations. This versatility constitutes a design advantage to maximize its applicability and power. While its roots go back to the early 90's, to the program originally authored by Estrin et al. (1993), with the first QM/MM applications on chemical reactivity dating from only a few years later (Elola et al., 1999), today *LIO* is a project in continuous evolution with a focus in performance, and the outcome of an interdisciplinary effort bringing together developers from chemistry and computer science backgrounds. *LIO* has been interfaced with the *Amber* package (Pearlman et al., 1995), to perform Born-Oppenheimer molecular dynamics and electron dynamics simulations in a hybrid QM/MM framework. In this context, the total energy is obtained according to the electrostatic embedding, additive QM/MM formulation:

(1) $$\begin{array}{l} E=E_{QM}+E_{MM}+E_{QM-MM} \end{array}$$

where the first term on the right hand side corresponds to the QM Kohn-Sham energy, the second one to the MM force field potential, and the third one is the coupling energy between the classical and quantum regions.

(2) $$\begin{array}{lll} E_{QM}\left[\rho \right] & = & T_{s}\left[\rho \right]+\sum_{I}\int \frac{\rho Z_{I}}{\left|\text{r}-{\text{R}}_{I}\right|}d\text{r}+\frac{1}{2}\int \int \frac{\rho \left({\text{r}}_{1}\right)\rho \left({\text{r}}_{2}\right)}{{\text{r}}_{1}-{\text{r}}_{2}}d{\text{r}}_{1}d{\text{r}}_{2}\\ +E_{xc}+\sum_{I}\sum_{A}\frac{Z_{I}Z_{A}}{{\text{R}}_{I}-{\text{R}}_{A}} \end{array}$$

(3) $$\begin{array}{lll} E_{QM-MM} & = & E_{LJ}^{QM-MM}\left(\left|{\text{R}}_{A}-{\text{R}}_{I}\right|\right)+\sum_{A\in MM}q_{A}\int \frac{\rho \left(\text{r}\right)}{\left|\text{r}-{\text{R}}_{I}\right|}d\text{r}\\ +\sum_{I\in QM}\sum_{A\in MM}\frac{Z_{I}q_{A}}{\left|{\text{R}}_{A}-{\text{R}}_{I}\right|} \end{array}$$

In the equations above, ρ represents the electron density, *T*_*s*_ the electronic kinetic energy, *E*_*xc*_ the exchange correlation term, *Z*_*I*_ the atomic number of quantum atom *I*, and *q*_*A*_ the partial charge of the classical atom *A*. $E_{LJ}^{QM-MM}$ is a non-electrostatic term, which describes dispersion and short range repulsion effects between QM and MM atoms, using Lennard-Jones potentials, consistently with the MM force field.

**Figure 1:**
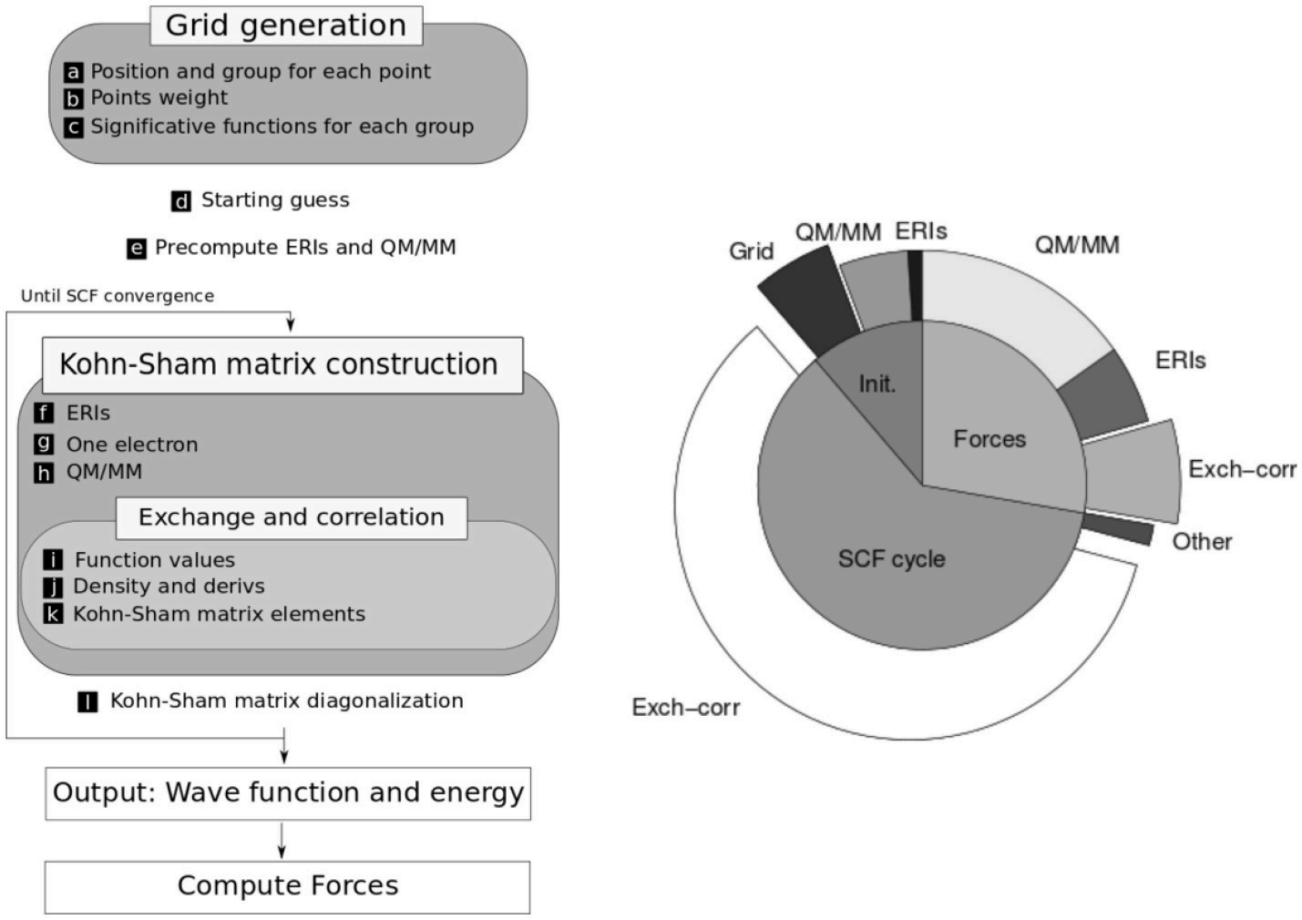
**(Left)** Scheme of the computation needed to perform a QM/MM molecular dynamics step. **(Right)** Relative times for one step of molecular dynamics simulation in a typical system with a serial algorithm (without GPU acceleration). Adapted with permission from Nitsche et al. (2014). Copyright 2014 American Chemical Society.

The kinetic energy and nuclear attraction contributions are calculated in terms of analytical one electron integrals, which are derived using Obara-Saika recursive equations (Obara and Saika, 1986). The electron repulsion term is computed in terms of two-electron repulsion integrals (ERI), which are also derived recursively. The exchange correlation energy is calculated using numerical spherically centered grids (Becke, 1988). The flow diagram of the computation scheme is depicted in Figure 1.

In the development of efficient algorithms for electronic structure calculations it is very important to consider the size of the systems to be treated. Hence, in *LIO* we put our focus on medium-sized systems (a few tens of atoms), which are the typical dimensions of the QM region in hybrid calculations. As can be seen in Figure 1, in this QM/MM scheme the major computational cost goes into the calculation of exchange-correlation, electron repulsion (ERIs) and QM/MM energy terms (in that order) and the corresponding forces contribution. In medium-size systems the cost of the computation is dominated by the exchange-correlation integral which scales linearly, whereas for larger systems the calculation of ERIs (which scale quadratically) and the diagonalization of the Fock matrix (which scales cubically) have an increasing importance.

**Figure 2:**
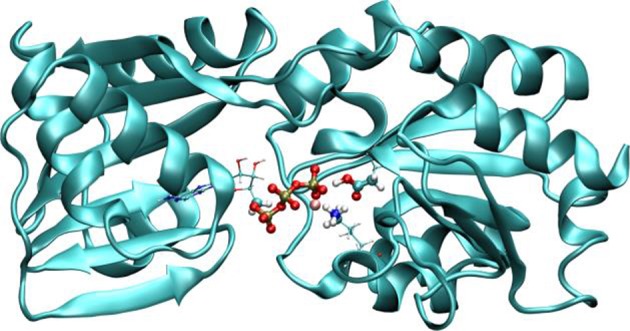
Representation of *Af*CopA QM/MM system. The QM region is defined by the atoms in the reactive region, which includes the phosphates of ATP, Mg^2+^, the aspartic 424 (which is phosphorylated) and part of the atoms of lysine 600. Adapted with permission from Nitsche et al. (2014). Copyright 2014 American Chemical Society.

Several optimization schemes were implemented to improve performance, like a linear scaling algorithm for exchange-correlation (Stratmann et al., 1996) or the use of auxiliary basis functions for the ERIs (Stratmann et al., 1996). In addition, these terms are totally or partially computed in the GPU (Nitsche et al., 2014). The overall result is a code that performs QM/MM molecular dynamics with high efficiency allowing for the computation of systems and/or properties at reduced computational costs. Table 1 shows the computation timings for some selected QM/MM systems discussed along the coming sections, and also for the case of the activation of copper-translocating P-type ATPase from *Archaeoglobus fulgidus* (*Af* CopA), an enzyme that couples the energy of ATP hydrolysis to catalyze Cu^+^ translocation across cellular membranes (see Figure 2) (Tsuda and Toyoshima, 2009).

**Table 1:** *LIO* performance in ground state molecular dynamics simulations, in terms of timings per QM/MM steps.

**System**	**QM atoms**	**MM atoms**	**Number of basis functions**	**Time per step (*LIO*) (s)**	**Time per step total (s)**	**Ps/day**
CH_3_S^−^+ H_2_O_2_	9	11445	79	0.622	0.74	113.9
*Mt*AhpE + H_2_O_2_	22	240000	178	1.55	3.86	22.4
*Af*CopA	34	61000	364	4.65	5.30	16.3

*Calculations were conducted in an Intel(R) Core(TM) i5-7400 CPU @ 3.00 GHz and a Gforce 980 TI GPU*.

Aside from standard molecular dynamics simulations, *LIO* can propagate the electron density as a function of time for a fixed molecular configuration, becoming, to the best of our knowledge, the first real-time TDDFT (RT-TDDFT) implementation in a QM/MM setting (Morzan et al., 2014). The evolution of the density matrix ρ is performed according to the Liouville-von Neumann equation:

(4) $$\begin{array}{l} \frac{\partial \rho }{\partial t}=\frac{1}{i\hbar }\left[H,\rho \right] \end{array}$$

where *H* is the Kohn-Sham matrix. The integration of this equation can be realized with either a Verlet integration scheme,

(5) $$\begin{array}{l} \rho \left(t+\Delta t\right)=\frac{2}{i\hbar }\left[H\left(t\right),\rho \left(t\right)\right]\Delta t+\rho \left(t-\Delta t\right) \end{array}$$

or the Magnus expansion to first order,

(6) $$\begin{array}{ll} \rho \left(t+\Delta t\right)= & \rho \left(t\right)-i\Delta t\left[H\left(t+\Delta t/2\right),\rho \left(t\right)\right]\\ -\frac{{\Delta t}^{2}}{2!}\left[H\left(t+\Delta t/2\right),\left[H\left(t+\Delta t/2\right),\rho \left(t\right)\right]\right]\\ +i\frac{{\Delta t}^{3}}{3!}\left[H\left(t+\Delta t/2\right),\left[H\left(t+\Delta t/2\right),\left[H\left(t+\Delta t/2\right),\rho \left(t\right)\right]\right]\right]+\ldots \end{array}$$

The advantage associated with the Magnus expansion is that it allows a greater Δt than the Verlet algorithm (usually in the order of 10 to 20 times larger), reducing the total number of steps needed for a fixed simulation time. On the other hand, the computational burden associated with the Magnus algorithm is much larger because it entails a greater number of matrix multiplications. However, these operations can be efficiently handled by GPUs, with a particularly high impact in the evaluation of the Magnus expansion which is usually truncated at order 10 to 50. Therefore, the use of the Magnus integrator turns out to be much more efficient than the use of the Verlet scheme when running on GPU.

The TDDFT scheme involves the evaluation of the Fock matrix, so all the optimizations made for SCF calculations are exploited here as well. Moreover, when working with fixed nuclear positions, other optimizations can be applied, especially in the QM/MM coupling integral and ERIs, significantly reducing their cost and leaving a quasi-linear scaling method. The efficiency of TDDFT depends on both the computation of one simulation step, and the maximum time step (Δt) that can be used. The later varies with the type of atoms in the QM zone and can be extended by using better propagators and/or freezing the inner electron density through the use of pseudopotentials. For a more detailed discussion please refer to Foglia et al. (2017). Table 2 illustrates the performance of TDDFT calculations comparing the full electron scheme with a pseudopotential approach.

**Table 2:** *LIO* RT-TDDFT performance for selected systems.

**System**	**Atoms**	**Full electron**			**ECP**			**Speedup**
		**Time Step (a.u.)**	**Average time per step (s)**	**fs/day**	**Time Step (a.u.)**	**Average time per step (s)**	**fs/day**	
CH_4_	5	0.076	0.022	7624	0.52	0.015	70500	9.25
CCl_4_	5	0.0095	0.030	680	0.16	0.022	15000	21.66
CI_4_	5	0.00075	0.059	24	0.16	0.022	15144	586.46
${\left[\text{Fe}{\left(\text{CN}\right)}_{6}\right]}^{4-}$	13	0.0038	0.141	48	0.0072	0.099	152	3.13
${\left[\text{Ru}{\left(\text{CN}\right)}_{6}\right]}^{4-}$	13	0.0011	0.178	17	0.018	0.108	348	20.58

*Effective core potentials and full-electron calculations were carried out with CEP and DZVP basis sets, respectively. Data from Foglia et al. (2017)*.

## Examples

### Chemical reactivity

The elucidation of the thermodynamics and kinetics of chemical reactions is one of the main goals of theoretical chemistry. In this context, the computation of free energy profiles assisted by QM/MM schemes has proved extremely useful to obtain mechanistic information (Kollman, 1993; Chipot and Pearlman, 2002; Hu and Yang, 2008; Carvalho et al., 2014). The two key ingredients for obtaining a meaningful free energy profile are the selection of the QM region and the QM level of theory on one hand, and the quality of the sampling on the other. Many sampling methodologies were developed and tested in order to optimize the tradeoff between cost and accuracy (Chipot and Pohorille, 2007). Each scheme presents advantages and caveats, but all of them rely in an extensive sampling of configurations of the system along the reactive process. The speedups described in the previous section, and the fact that *LIO* can be coupled to different MD engines, like *Gromacs* (Van Der Spoel et al., 2005) or *Amber* (Pearlman et al., 1995), allow us to attain this kind of sampling at the DFT level. It is worth noting that obtaining each of these profiles often requires ~0.5–1 ns of QM/MM MD sampling. The following sections show selected examples of chemical reactions studied with our code, both in aqueous solution and in enzyme catalyzed processes. In these particular cases, the QM region computations were performed at the generalized gradient approximation (GGA) using the *PBE* combination of exchange and correlation functionals, with a dzvp basis set (Godbout et al., 1992). The MM region was treated with the *Amber99* forcefield (Lindorff-Larsen et al., 2010).

#### Reactivity in aqueous solution: nitrous oxide formation upon nitroxyl decomposition

Modeling solvent effects on chemical reactivity, and the influence of aqueous solvation in particular, has been among the main goals of computational chemistry, since much of the most relevant processes in chemistry, biochemistry and materials sciences, take place in solution or at a solid-liquid interface. Here we illustrate the application of our code to aqueous chemistry through an example involving nitroxyl, a key species in redox biochemistry. Nitroxyl (HNO) is a species playing different roles in nitrosative stress processes with great interest in the pharmacology field due to its potential use in heart failure treatment, as well as its vasodilator properties and its role in cellular metabolism (Ma et al., 1999; Miranda, 2005; Miranda et al., 2005). Nitroxyl rapidly decomposes in aqueous solution yielding nitrous oxide (N_2_O) (Shafirovich and Lymar, 2002). This is a fast reaction that competes with many other chemical processes in which HNO may be involved in a cellular context. Then, a detailed molecular description of this phenomenon is of general interest in biochemistry.

Great efforts have previously been done from an experimental and theoretical point of view to study the different steps leading to HNO decomposition (Shafirovich and Lymar, 2002; Fehling and Friedrichs, 2011). It was proposed that the mechanism involved a dimerization of HNO followed by a cleavage of one of the N-O bonds, to yield N_2_O and water. Our aim was to fully characterize the energetics and the molecular determinants of the reaction mechanism, taking into account the influence of the environment, which could be extremely important especially because an acid-base equilibrium was proposed to be involved in the mechanism. Therefore, we studied both steps of the reaction mechanism, evaluating different protonation states and possible isomers, by means of multiple QM/MM MDs, using the umbrella sampling scheme to determine the corresponding free energy profiles (Bringas et al., 2016).

**Figure 3:**
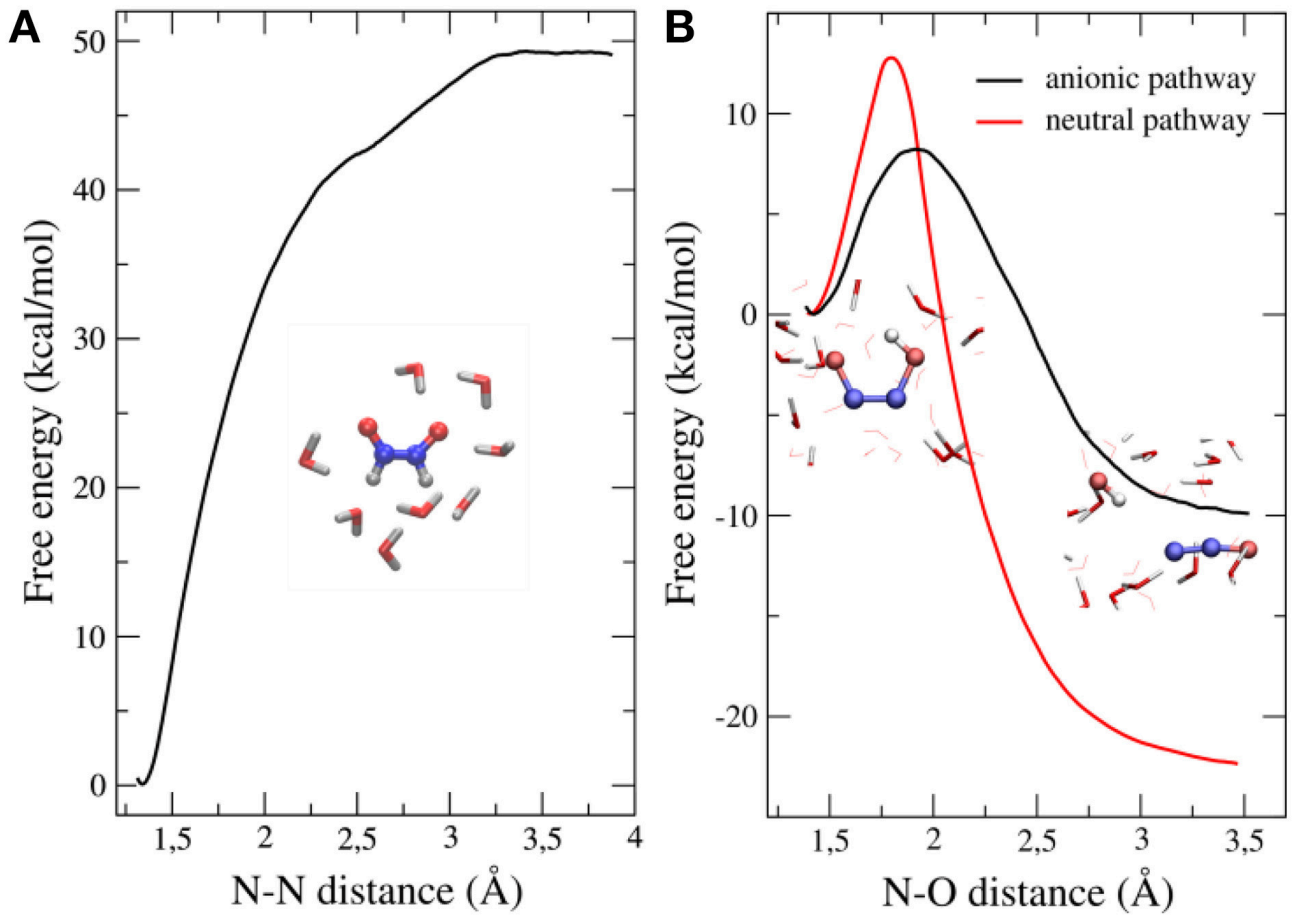
Free energy profiles of both steps leading to N_2_O formation starting from HNO dimerization. **(A)** Dimerization profile. **(B)** N_2_O formation profile. Results for both anionic and neutral pathways are shown. Adapted from Bringas et al. (2016), with permission from Elsevier.

Our results showed that the dimerization is an exergonic process that occurs without a significant activation barrier (Figure 3A), and that the *cis* isomer of the dimer (HONNOH) is more stable than the *trans* one by ~2 kcal/mol (data not shown). The dimer intermediate might be involved in different acid-base equilibria, so we investigated the second step of the reaction, starting from different protonation states (Figure 3B). The anionic path showed a much lower activation barrier (~7 kcal/mol) than the one corresponding to the neutral path (~14 kcal/mol). This effect is mainly explained by a more stable transition state for the anionic pathway, due to specific interactions with water molecules (Bringas et al., 2016).

The available experimental data and this analysis allowed us to propose an overall reaction mechanism for HNO decomposition that can be written as a consecutive reactions scheme with quick acid-base equilibria connecting products and reactants of different steps:

(7) $$\begin{array}{r} 2HNO\overset{k_{1}}{\to }cis-ON\left(H\right)N\left(H\right)O\\ cis-ON\left(H\right)N\left(H\right)O⇆cis-HONNOH\\ cis-HONNOHD⇆{\left[cis-HONNO\right]}^{-}+H^{+}\\ cis-HONNOH\overset{k_{2}^{NP}}{\to }N_{2}O+H_{2}O\\ {\left[cis-HONNO\right]}^{-}\overset{k_{2}^{AP}}{\to }N_{2}O+HO^{-} \end{array}$$

where *k*_1_, $k_{2}^{NP}$, and $k_{2}^{AP}$ are the constants for the dimerization and for the dissociation via the neutral and anionic pathway elemental steps, respectively. Using transition state theory and the predicted *pK*_*a*_s for the dimer intermediate (Fehling and Friedrichs, 2011), we estimated that the anionic pathway will be the only one operative near physiological pH.

In summary, the detailed molecular description of the reaction mechanism at the QM/MM level, showed that specific interactions between the reactive species and the water molecules turn out to be determinant in the stabilization of transition states, thereby modifying the free energy barriers. We predicted a strong pH-dependence of the overall kinetics of N_2_O formation, related with the fraction of reactive species available in solution, in agreement with previous experimental data.

#### Protein catalysis

The investigation of the molecular basis of enzyme catalysis has attracted the attention of many researchers both in the experimental as well as the computational sides. To illustrate the application of our code to this objective, we have chosen hydroperoxides reduction as case study. The reduction of cellular endogenous or exogenous hydroperoxides is a key biochemical reaction associated not only with the cellular redox homeostasis, but also with signaling and regulation processes (Hopkins, 2017). One of the most important antioxidant mechanisms in biological systems is the reduction of peroxides through the oxidation of low molecular weight thiols (LMW) and/or reactive cysteine (Cys) residues in proteins:

(8) $$\begin{array}{l} RS^{-}+R\prime OOH\to \;RSO^{-}/RSOH+R\prime OH/RO^{-} \end{array}$$

where the reactive species are the thiolate and the hydroperoxide. Although the reaction of peroxides with LMW thiols is usually slow (~10 M^−1^ s^−1^ for H_2_O_2_) (Winterbourn and Metodiewa, 1999), some enzyme thiols react several orders of magnitude faster in terms of second order rate constants. Among these Cys-based peroxidases, peroxiredoxins (Prx) are the most significant ones, given their reactivity, distribution, and concentration (Hofmann et al., 2002; Winterbourn, 2008).

**Figure 4:**
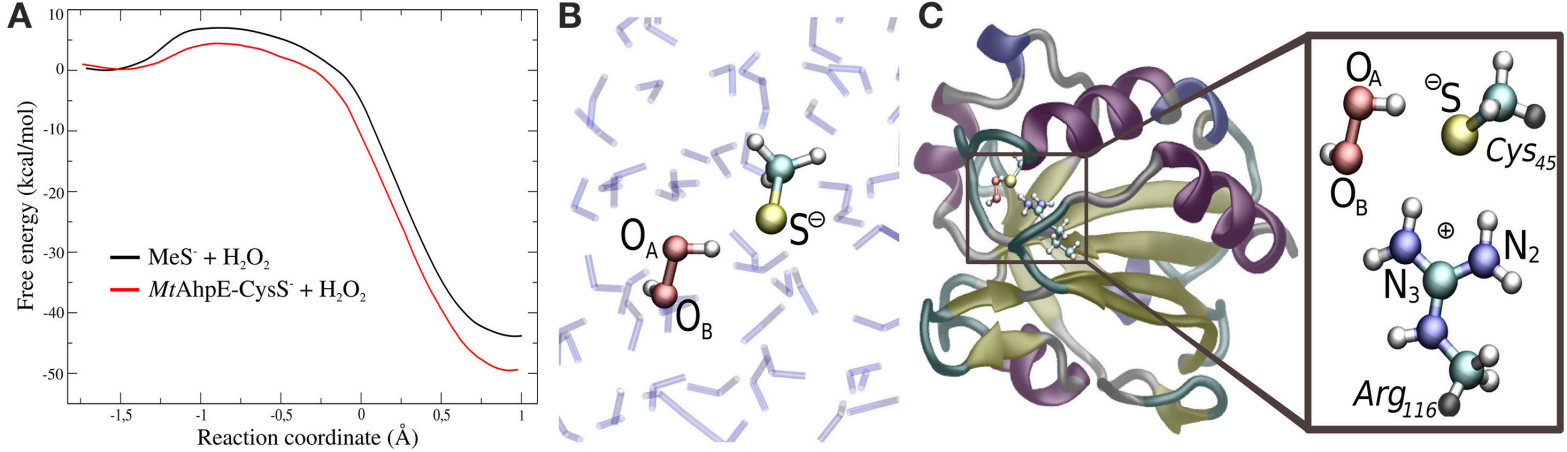
Comparison of the reactivity of thiols against that of peroxides in aqueous solution and in the Prx's active site. **(A)** Free energy profiles for both systems. **(B,C)** QM subsystems for the reaction in water **(B)** and in *Mt*AhpE **(C)**. Modified with permission from Zeida et al. (2012, 2014). Copyright 2012 American Chemical Society.

In recent years, we have applied QM/MM techniques to shed light on the molecular determinants that govern this extremely important biochemical reaction, comparing the reactivity of LMW thiols with Prx, and assesing also different hydroperoxides (Zeida et al., 2012, 2013, 2014, 2015). In particular, aiming to gain microscopic insight onto the Prxs active site's properties that could explain the catalytic effect of these systems, we performed QM/MM MDs to determine the free energy profiles of reaction 8 for H_2_O_2_, with the thiolate being CH_3_S^−^ or the reactive Cys of the alkyl hydroperoxide reductase E from *Mycobacterium tuberculosis* (*Mt*AhpE), the 1-Cys Prx of the mycobacteria genome as a Prx model (Zeida et al., 2012, 2014). The umbrella sampling approach was applied, choosing the reaction coordinate as the difference between the O_A_-O_B_ and S-O_A_ distances (Figure 4A). We have also measured the activation thermodynamics parameters of the catalyzed reaction by means of temperature dependence stopped-flow kinetics experiments and the Eyring's formalism (Table 3).

**Table 3:** Experimental kinetic and thermodynamics activation parameters for the reduction of H_2_O_2_ by free Cys and *Mt*AhpE.

	***k*_2_ (M^−1^ s^−1^)^a^**	***E*_act_ (kcal/mol)**	**Δ*H*^†^ (kcal/mol)**	**Δ*S*^†^ (cal/K.mol)**	**Δ*G*^†^ (kcal/mol)**
Free Cys^b^	14.9	17.0	16.4 (0.3)	1.7 (1.1)	15.9
*Mt*AhpE-Cys_45_^c^	8.2 × 10^4^ (1.5)	5.7 (0.7)	4.8 (0.5)	−19.1 (1.9)	10.5

*Standard deviations in parenthesis when available*.

a *pH-independent rate constants*.

b *data taken from Luo et al. (2005)*.

c *data taken from Zeida et al. (2014)*.

Figure 4A shows the energy profile for the uncatalyzed and catalyzed processes, while Figures 4B,C provide a schematic representation of the QM subsystem for each case. The corresponding Δ*G*^†^s of ~8 and ~4 kcal/mol turn out to be underestimated in comparison with the one determined experimentally (see Table 3), which can be possibly attributed to the flaws of DFT at the GGA level for determining activation energies (Zhao and Truhlar, 2008). However, the catalytic effect, meaning the difference between the activation free energies (ΔΔ*G*^†^), is about ~4 kcal/mol, consistently with the experimentally determined ΔΔ*G*^†^ = 5.4 kcal/mol and with the ~5000-fold increase in reactivity observed (Luo et al., 2005; Hugo et al., 2009).

It is worth noticing that these profiles are not exactly the same as those published earlier (Zeida et al., 2012, 2014), due to improvements in our computing capabilities and significant advances in sampling protocols. Both new profiles display lower free energy barriers, which is expected for better explorations of the free energy landscape. Nevertheless, the reaction mechanism and properties along the reaction coordinate do not show significant changes, and, most importantly, the ΔΔ*G*^†^ remain unaffected, and so the catalytic effect is still being reproduced in fair agreement with the experimental data.

The exploration of the reaction coordinate allow us to identify key events during the reaction to explain the differences in the Δ*G*^†^s. Specifically, the strong interactions of the thiolate and the peroxide with Arg_116_ and Thr_42_ residues, which are extremely conserved among the Prx family (Soito et al., 2011; Perkins et al., 2015), are the main factors responsible for the transition state stabilization and the concomitantly significant reduction in Δ*H*^†^, which in turn results in a decrease in Δ*G*^†^ in spite of the unfavorable entropic contribution (Table 3). Our calculations support the idea of a bimolecular nucleophilic S_N_2 type substitution mechanism, with an internal proton transfer and no acid-base catalysis. The catalytic ability of Prxs lies on the stabilization of the transition state due to an active site design that configures a complex H-bond network activating both reactive species, the thiolate and the peroxide.

### Spectroscopic studies

UV-vis or FTIR spectrophotometers are ubiquitous tools in research laboratories, routinely employed to characterize new species, to establish the identity of a chemical systems, to perform both thermal and photochemical reactivity studies, or even to analyse the presence of impurities in a given sample. Ambiguities in the characterization of unknown species are often present, and so experimental chemists are increasingly relying on theoretical calculations to complement their measurements.

The vast majority of the computational studies addressing electronic or vibrational spectra involve the geometry optimization of the molecule at a given level of theory, and the study of its spectroscopic properties at those frozen nuclear coordinates. Within this framework, the emulation of the environment is carried out by different approaches, as the Polarizable Continuum Model (PCM) (Tomasi et al., 2005), often placing a few explicit solvent molecules in some fixed position in the first solvation shell interacting with the chromophore (Zuehlsdorff et al., 2016) or, more recently, using a classical and explicit description of the solvent by employing a QM/MM hybrid Hamiltonian (Barone et al., 2010). The most common approach to calculate infrared spectra is the harmonic oscillator approximation, through the diagonalization of the Hessian matrix at different levels of theory. This process yields the vibrational modes, their characteristic frequencies, and an estimation of the intensity of those bands in the infrared spectrum (Bloino et al., 2016). However, this scheme may present flaws in cases in which there are strong anharmonicities or specific solute-solvent interactions. The IR spectrum can also be computed directly from a molecular dynamics simulation including explicit solvent molecules. The absorption spectrum can be determined as the Fourier Transform of the temporal autocorrelation function of the dipole moment (Futrelle and McGinty, 1971; McQuarrie, 1976):

(9) $$\begin{array}{l} I\left(\omega \right)=\frac{1}{2}\int dte^{i\omega t}\langle \overset{⇀}{\mu }\left(0\right)|\overset{⇀}{\mu }\left(t\right)\rangle \end{array}$$

In the previous equation, the brackets denote an equilibrium ensemble average, which can be obtained from molecular dynamics simulations:

(10) $$\begin{array}{l} \langle \overset{⇀}{\mu }\left(0\right)|\overset{⇀}{\mu }\left(t\right)\rangle \approx \frac{t-t_{i}}{\Delta t}\sum_{i}\overset{⇀}{\mu }\left(t_{i}\right)\overset{⇀}{\mu }\left(t_{i}+t\right) \end{array}$$

where Δ*t* is the integration time-step. Similarly, the Fourier Transform of the temporal autocorrelation function of the polarizability yields the Raman spectrum of a given system.

In the case of the electronic spectra, Time Dependent Density Functional Theory in the linear response formulation (LR-TDDFT) has become in the last two decades the most popular approach to predict the electronic excitation frequencies of middle-size systems, due to its modest computational cost and relatively good performance in comparison with highly correlated schemes (Runge and Gross, 1984; Marques et al., 2006). Alternatively, it is also possible to compute an electronic spectrum from electron dynamics simulations, through real-time TDDFT. This methodology, which is incorporated in *LIO*, is not so commonly used to calculate electronic frequencies. This is related to the fact that the electron dynamics simulations needed to recover the absorption frequencies with RT-TDDFT require to excite and propagate the density matrix of the electronic system for tens or hundreds of femtoseconds, and then perform an *ad-hoc* post-processing analysis. This scheme tends to be more demanding, and somehow prevents RT-TDDFT from being used in a black-box format. However, the real-time approach exhibits some appealing features in comparison with LR-TDDFT, that include the possibility to study intense perturbations beyond the linear response regime (Marques et al., 2006; Lopata and Govind, 2011), the scaling of the computation burden with respect to the number of electrons, that can be made quasi-linear, or the avoidance of the computation of the exchange-correlation kernel. Therefore, RT-TDDFT could be a competitive alternative to the linear response method, especially in the case of large systems, since the computation time in typical LR-TDDFT implementations grows as *N*^3^ or *N*^4^.

Beyond the applied methodology and the level of sophistication of the electronic structure calculations, one of the main causes of the failures in the prediction of spectroscopic properties are due to the consideration of a frozen nuclear geometry, obtained by an initial optimization, thus neglecting thermal fluctuations. A proper exploration of configurational space produces spectra averaged on the degrees of freedom of both the chromophore and its environment, providing smooth lineshapes instead of the discrete, multiple-lines spectra corresponding to the particular transitions energies obtained from a single geometry. The use of molecular dynamics or Monte Carlo simulations where the spectra are obtained from statistical averages within an ensemble has become an increasingly chosen strategy for different types of systems (Valsson et al., 2013).

#### Vibrational spectra of simple aqueous species: early results and benchmarks

**Figure 5:**
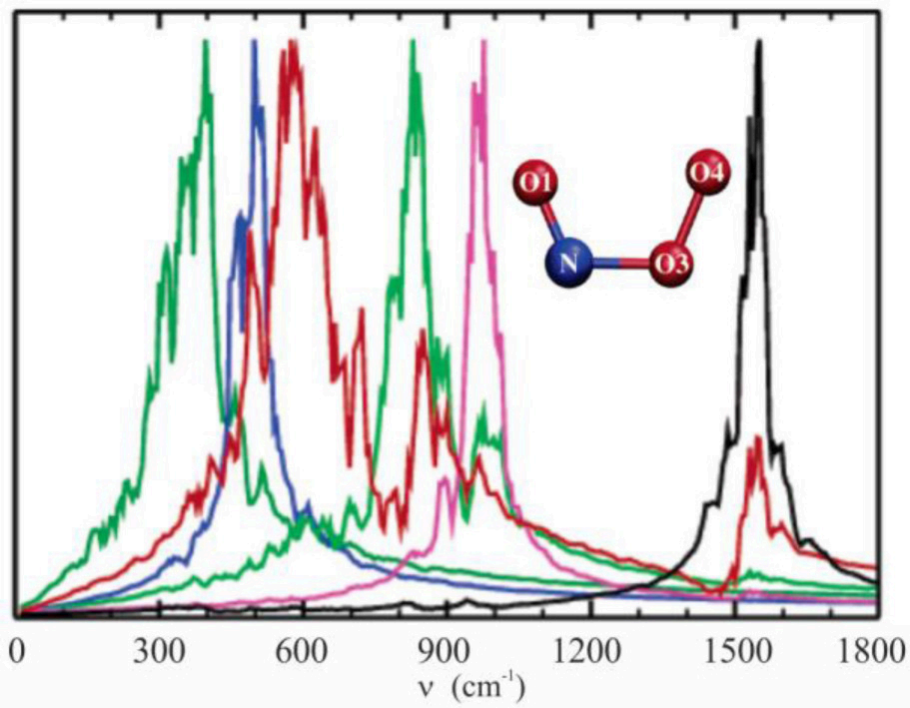
Vibrational density of states computed as the Fourier transform of the velocity autocorrelation function, evaluated in the isolated species normal modes coordinates. Molecular dynamics simulations were done with the PBE functional and the TIP4P model for water. NO1 stretching, black line; O1NO3 bending, pink line; O3O4 stretching, green line; NO3 stretching, red line; OONO torsion, blue line; NO3O4 bending, dark green line. Adapted with permission from Gonzalez Lebrero et al. (2005). Copyright 2005 American Chemical Society.

In 2005 we investigated the structure and the vibrational spectrum of peroxynitrite anion in solution via QM/MM molecular dynamics simulations, calculating the temporal autocorrelation functions of the atomic velocities (Gonzalez Lebrero et al., 2005). At that time, the computation capabilities allowed us to perform production dynamics of 20 ps in a reasonable time window. This study provided a picture of the complex interactions between the ONOO^−^ anion and the solvent, and how these were reflected in the vibrational spectrum in solution, as shown in Figure 5. Our results yielded frequency values much closer to the experimental ones than those obtained using standard methodologies, and also helped to assign a controversial band centered at 642 cm^−1^ as corresponding to NO3 stretching.

**Figure 6:**
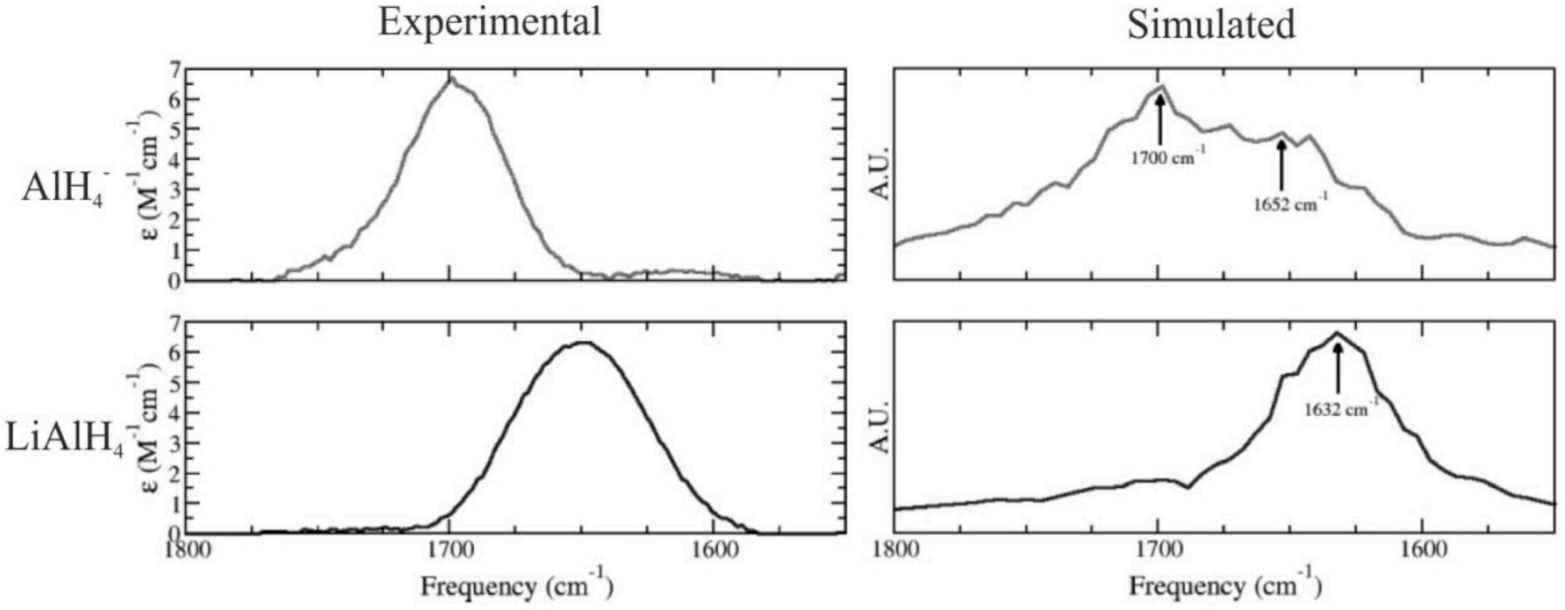
Simulated **(Left)** and experimental **(Right)** infrared spectra for the ${\text{AlH}}_{4}^{-}$ anion (top) and LiAlH_4_ ionic pair (bottom) in ethereal solution. Adapted with permission from Bikiel et al. (2005). Copyright 2005 American Chemical Society.

The nature of the solute species present in ethereal solutions of LiAlH_4_ is of crucial importance to understand the mechanism for the reduction of ketones and other functional groups by LiAlH_4_. In 2005, we have employed a combination of experimental and theoretical techniques to investigate the structure of this system in ethereal solutions, using QM/MM simulations in which LiAlH_4_ was modeled at the DFT PBE level using dzvp basis sets, and the solvent was described using a three site potential (Bikiel et al., 2005). Our results were consistent with a dissociation equilibrium displaced to the associated species. However, a significant amount of the dissociated species is also expected to exist. We calculated the infrared spectra for both LiAlH_4_ and ${\text{AlH}}_{4}^{-}$ species performing molecular dynamics simulations, reproducing the main features of the experimental spectra, as shown in Figure 6.

#### The electronic spectrum and the sampling issue

In 2014 we have developed a powerful scheme to perform real-time TDDFT electron dynamics in a QM/MM framework (Morzan et al., 2014). This implementation can easily handle quantum subsystems in the order of 100 atoms surrounded by thousands of classical nuclei, enabling the investigation of the effect of a complex environment, such as a solvent or a protein matrix, on the UV-vis spectra of molecular systems. Our starting point was the validation on isolated species by comparison of the absorption maxima obtained using the real time and the linear response methodologies (some of these results are presented in Table 4). These molecules were chosen as a benchmark to test our code and verify that the obtained spectra were in very good agreement with experimental results.

**Table 4:** Calculated and experimental absorption maximums (λ_max_ (nm)) of different molecules.

**Compound**	**RT-TDDFT**	**LR-TDDFT**	**Experimental**
H_2_O	161	159	170
CO	147	143	145
C_6_H_6_	175	172	180
Tryptophan	298	311	286

*Real-time and linear-response TDDFT calculations were performed with the PBE exchange-correlation functional and dzvp basis set*.

The effect of the environment becomes crucial within proteins and here is where the QM/MM methodologies make the difference. In the work mentioned above, the spectrum of the CO hexa-coordinated heme group in Flavohemoglobin of *Escherichia Coli* (*Ec*FlavoHb) was analyzed. The results showed that the Soret band of the chromophore experiences a notorious blue shift (Δλ~35 nm) when going from vacuum to the protein environment. Despite the lack of a direct experimental comparison, the observed shift in the *Ec*FlavoHb heme with respect to the gas phase is in accordance with the expected trend.

**Figure 7:**
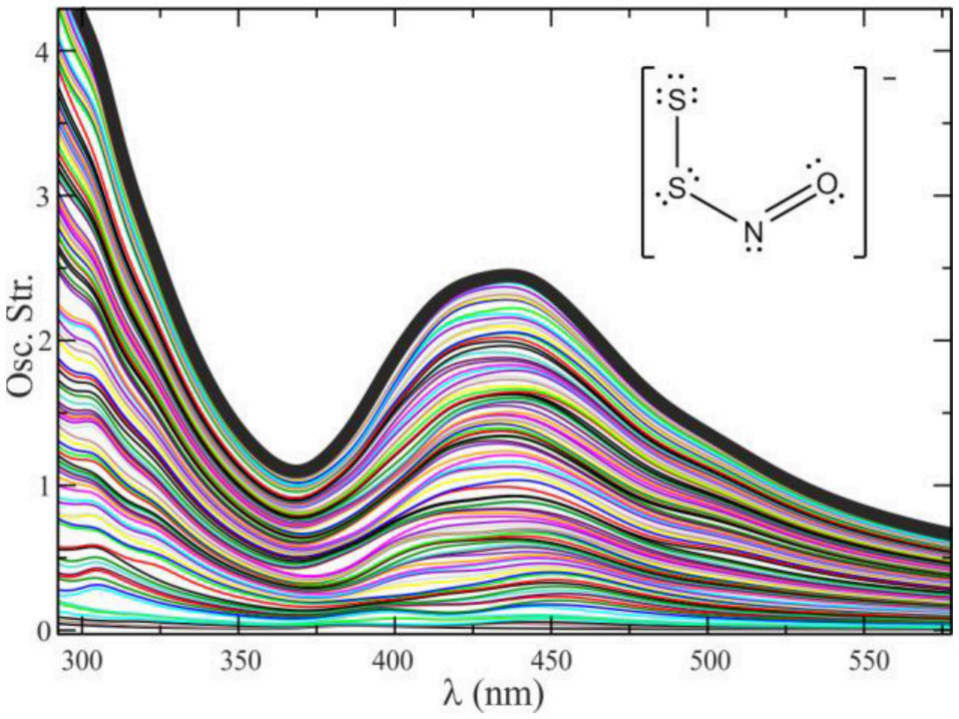
Convergence process for the spectrum of S_2_NO^−^ in methanol, by averaging 110 different configurations. The global spectrum is calculated as $E\left(\lambda \right)=\frac{1}{N}\overset{N}{\underset{j=1}{\sum }}e_{j}\left(\lambda \right)$, where *e*_*j*_(λ) represents the spectrum for a given geometry *j*.

The examples given so far correspond to electronic spectra extracted from electron dynamics simulations on a single geometry. To generate a realistic electronic spectrum that considers thermal effects, we calculate the spectra of a set of configurations extracted from a QM/MM molecular dynamics trajectory. These configurations must be separated from one another by a time frame η long enough as to decorrelate the molecular vibrations (for simple molecular systems in solution it is usually enough with η = 5–10 ps). The number of configurations needed to get a converged spectrum in solution is typically of a few dozens. A healthy practice is to combine, i.e., to intercalate, the QM/MM molecular dynamics simulations with some purely classical sampling, especially in systems with many degrees of freedom and multiple solvation structures, to amplify the exploration and avoid the system to get trapped around a local minimum. Figure 7 shows a typical convergence sequence in the computation of an electronic spectrum using QM/MM dynamics.

As seen in Figure 7, it is of a huge importance to perform an adequate sampling of the nuclear configurations visited along the dynamics to obtain a spectrum with the absorption maximum located in the correct position and with the correct band shapes. Unfortunately, there is no magic number associated with the required amount of nuclear configurations to reach convergence. This number is usually tied to several factors, mainly the rigidity of the molecular system under study (and therefore the number of accessible local minima) and the magnitude and lability of the interactions between the solute and the solvent.

**Figure 8:**
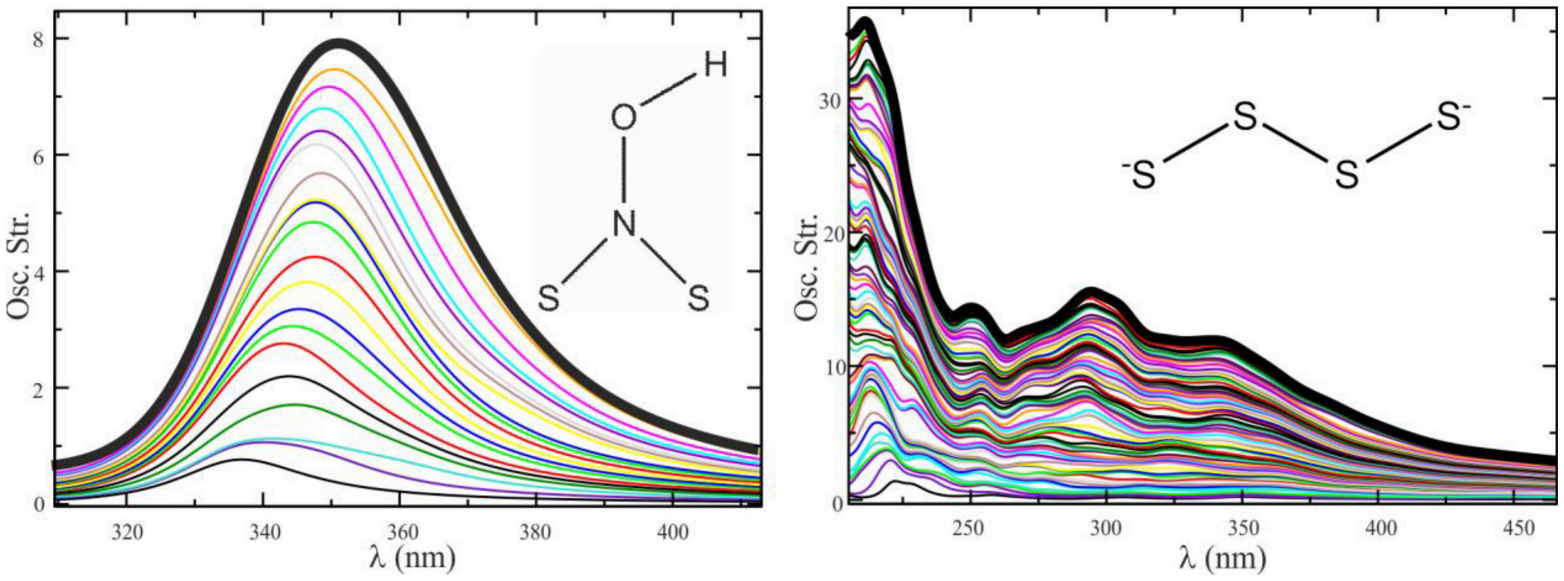
Convergence sequence for the spectrum of (HO)NS_2_ in acetonitrile **(Left)** and ${\text{S}}_{4}^{2-}$ in water **(Right)**. Ten configurations provide a converged profile in one case, whereas more than 70 do not seem enough in the other.

Figure 8 shows that the convergence of the electronic spectrum of the (HO)NS_2_ molecule in acetonitrile is achieved by averaging less than 10 spectra taken at snapshots separated by 5 ps of QM/MM dynamics, while for more flexible species like ${\text{S}}_{4}^{2--}$ (with high rotational freedom) said process is not satisfactorily converged after averaging 70 spectra.

Ensuring that a spectrum is statistically converged is not only crucial to find the correct values of λ_max_; it is also important because, if not converged, the spectra may present shoulders similar to those found in experiments, thus leading to incorrect interpretations.

#### Solving specific questions: the case of the “crosstalk” between NO and H_2_S

We will illustrate the capabilities of our code, by showing results related to the crosstalk chemistry between NO and H_2_S. In addition to the diverse biological roles of nitric oxide (NO) and hydrogen sulfide (H_2_S), there is a growing appreciation that both molecules have interdependent biological actions resulting in either mutual attenuation or potentiation responses, the so-called NO/H_2_S “cross-talk” (Marcolongo et al., 2017).

The study of the interaction pathways between these two molecules has led to a large number of publications throughout this decade, and there have been many controversies around the appearance and assignation of spectroscopic signals in different experiments. In particular, there has been much debate around the appearance of a transient signal at ~409 nm produced in the reaction of S-nitrosoglutathione (GSNO) with HS^−^ at pH = 7.4 (Filipovic et al., 2012). Filipovic and colleagues argue that this signal corresponds to the presence of a mixture of polysulfides while other groups claim that the yellow signal corresponds to perthionitrite (S_2_NO^−^), the sulfur analog of peroxynitrite (Seel and Wagner, 1988; Munro and Williams, 2000; Filipovic et al., 2012; Cortese-Krott et al., 2015; Bailey et al., 2016; Bolden et al., 2016). This anion has been well-characterized taking part of solid salts and in organic solvents (Seel et al., 1985; Filipovic et al., 2012; Wedmann et al., 2015), though their clear identification and chemistry in aqueous solutions remains elusive.

In 2016, we tested the use of QM/MM dynamics in conjunction with the application of RT-TDDFT implemented in *LIO* to try to help in the elucidation of this controversial signal, tilting the balance toward the proposal of SSNO^−^ as the responsible for the yellow signal (Marcolongo et al., 2016). Our calculations for this species (together with a large number of related benchmark molecules) showed that SSNO^−^ is a good candidate to absorb in that region and also reproduces quantitatively the solvatochromic shift of the absorption band while going from water to a set of organic solvents where the species is well-characterized. The QM/MM dynamics performed by our group showed that the specific interactions between SSNO^−^ and the solvent are responsible for the modulation of the θ(N_1_-S_1_-S_2_) angle which strongly affects the spectroscopic properties of this molecule, as shown in Table 5. For these simulations, a TIP3P potential was utilized to describe the water molecule and the force fields for acetonitrile, acetone and methanol were generated following the restricted electrostatic potential (RESP) technique and DFT calculations at the PBE/*dzvp* level. Equilibrium distances and angles, as well as force constants were computed using the same electronic structure scheme.

**Table 5:** Calculated and experimental absorption maximums for S_2_NO^−^ in different solvents, as well as the mean value of the θ(N_1_-S_1_-S_2_) angle.

**Solvent**	**θ(N_1_-S_1_-S_2_) (°)**	**λ_max_ (nm) (experimental)**	**λ_max_ (nm) (RT-TDDFT)**	**Number of averaged spectra**
Water	111	409–412	411	54
Acetone	113	448	458	43
Acetonitrile	117	450	458	63
Methanol	117	431	425	110

The results obtained for this system showed how the statistical study of the interactions resulting from the QM/MM sampling is the key to obtain results that can be compared to the experimental values almost quantitatively.

## Concluding remarks and perspectives

In this article, we have provided an overview on the basic features of the *LIO* program, focusing on some representative applications. This review has emphasized the fundamental importance of the code performance in the study of chemical reactivity and free energy profiles, and in the simulation of vibrational and electronic spectra. Currently, most QM/MM DFT programs are capable of handling molecular systems having, in the quantum and classical domains, in the order of a few hundreds and a few thousands of atoms, respectively. However, the computational cost associated with systems of this size restrains their applicability to single-point calculations or partial geometry optimizations, which may be helpful to analyze some structural or energetic aspects at zero temperature, whereas the kind of applications considered in this article require extensive sampling. A data point along a reaction coordinate profile, or the construction of an auto-correlation function to extract a vibrational spectrum, typically require molecular dynamics simulations in the order of at least several picoseconds. To reach these time-scales, the cost per iteration cannot exceed a few seconds. The goal in developing the *LIO* program is not to save computation time in the calculation of a given property, but to make it feasible when it was not. Thanks to recent advances in the code, it is currently possible to study models containing about 30 QM atoms in the quantum region for time windows in the order of the nanosecond, using a single commercial GPU.

In *LIO*, the most expensive parts of the QM/MM DFT scheme, including the calculation of the exchange-correlation energy, Coulomb interactions, and forces, have been thoroughly optimized, reaching an almost linear-scaling performance. As a consequence of these improvements, those operations typically inexpensive start to become the new limiting steps in the overall speed. One example of this is the diagonalization of the Hamiltonian, which, except in the case of very large systems, takes up a negligible fraction of the SCF iteration cost in Gaussian basis codes. At the present stage, diagonalization, which scales as *N*^3^, represents the next potential bottleneck for big systems. For this reason, *LIO* is not a “linear-scaling” implementation, although it does behave approximately linearly within a certain size range, when the diagonalization still does not contribute appreciably to the computational load. However, the diagonalization dominates the overall performance in the case of very large systems exceeding 200 or 300 atoms in the quantum region. Thus, to go beyond these sizes, the implementation of an order *N* diagonalization algorithm should be made available.

As discussed in the Examples section, a linear-scaling behavior is particularly appealing for the computation of electronic spectra of large molecules. Presently, most UV-visible spectroscopy calculations involve TDDFT in the linear response formulation. This implementation, typically scaling as *N*^3^ or *N*^4^, is often easier to use in comparison with the real time approach, and more efficient than this one for systems of small and moderate size. Nevertheless, for large systems in complex environments, real-time TDDFT simulations with an approximately linear scaling might be a smart alternative to surpass the size limitations of the linear response method. Besides, the feasibility to sample the structural degrees of freedom in solution or in a biomolecule with the same Hamiltonian employed for the electron dynamics, constitutes an asset of the *LIO* code.

Aside from spectroscopic applications, real-time TDDFT offers the possibility to perform quantum transport simulations. Recently, we have implemented in *LIO* an approach to compute molecular conductance in open quantum conditions (Morzan et al., 2017). The combination of RT-TDDFT with a QM/MM framework provides a unique platform to investigate challenging phenomena related to electron dynamics in realistic environments, which are very difficult to address from the experimental side or even with other modeling strategies. In our group, we are studying the electron transfer dynamics to and from the CuA active site in cytochrome C oxidase. Charge transport between redox sites in an enzyme is a fascinating phenomenon that might be studied with our approach, providing that the number of atoms involved in the likely paths foreseen for the electrons is not excessive to be treated within the QM region. The conductance of conjugated polymers in a disordered matrix, or the electron exchange at an electrochemical interface, are also problems that could be explored using the present approach, where the solvent, the counterions, or, more generally, the surrounding media, can be described at the MM level.

The use of pseudopotentials, recently made available in the code (Foglia et al., 2017), is fundamental to perform real time TDDFT simulations with transition metals and heavy atoms. The time-propagation of the core electrons, with high characteristic frequencies, demands integration time-steps that may be orders of magnitude below those required for the integration of the valence density. Thus, all-electron TDDFT dynamics with transition metals or atoms below the second period are extremely costly because of the small time-steps necessary for energy conservation. The incorporation of pseudopotentials has relaxed this constraint, making it feasible the electron dyamics simulations of the active sites of metalloenzymes or metallic wires. The pseudopotential implementation of the forces, necessary to perform MD simulations, is currently underway.

To summarize, the *LIO* code is a very active project and a valuable resource, open to the community via github (https://github.com/MALBECC/LIO), to perform DFT molecular dynamics and real time TDDFT simulations in a QM/MM framework. Beyond those objectives concerning performance, which have concentrated much of the efforts of recent years, there is presently a strong drive toward the development of new capabilities. Some of the features or implementations currently in progress are:

- the linear response TDDFT method;
- pseudopotential forces;
- intensities in resonant Raman spectra;
- exact exchange terms for hybrid DFT in GPU;
- schemes to compute quantum transport;
- Ehrenfest dynamics;
- coupling with libxc (Marques et al., 2012) in order to extend the variety of available DFT functionals.

Hopefully, this review has made it clear how much realistic chemical and spectroscopic predictions rely on sampling efficiency, necessary to introduce both thermal and environment effects. This is the spirit that has guided the evolution of *LIO*, which has been optimized for QM/MM simulations where the typical size of the QM domain reaches a few tens of atoms. Perhaps the most important challenge for the development of molecular simulation methods is to keep up with the advances in HPC platforms, algorithmic optimization, and theory. To take the proper advantage from all these worlds is only possible through a collaborative endeavor involving theoretical chemists and computer scientists, with the continuous feedback from the experimental side.

## Author contributions

AZ, JM, JS, NF, and UM selected and analyzed the data. AZ, JM, MG, DE, and DS wrote the paper. MG, DE, and DS supervised research.

### Conflict of interest statement

The authors declare that the research was conducted in the absence of any commercial or financial relationships that could be construed as a potential conflict of interest.
